# Detecting Deceptive Dark-Pattern Web Advertisements for Blind Screen-Reader Users

**DOI:** 10.3390/jimaging9110239

**Published:** 2023-11-06

**Authors:** Satwik Ram Kodandaram, Mohan Sunkara, Sampath Jayarathna, Vikas Ashok

**Affiliations:** Department of Computer Science, Old Dominion University, Norfolk, VA 23529, USA; skoda004@odu.edu (S.R.K.); msunk001@odu.edu (M.S.); sampath@cs.odu.edu (S.J.)

**Keywords:** extraneous content, deceptive adverts, ad blockers, dark patterns, blind users, screen readers, multi-modal classification, accessibility

## Abstract

Advertisements have become commonplace on modern websites. While ads are typically designed for visual consumption, it is unclear how they affect blind users who interact with the ads using a screen reader. Existing research studies on non-visual web interaction predominantly focus on general web browsing; the specific impact of extraneous ad content on blind users’ experience remains largely unexplored. To fill this gap, we conducted an interview study with 18 blind participants; we found that blind users are often deceived by ads that contextually blend in with the surrounding web page content. While ad blockers can address this problem via a blanket filtering operation, many websites are increasingly denying access if an ad blocker is active. Moreover, ad blockers often do not filter out *internal* ads injected by the websites themselves. Therefore, we devised an algorithm to automatically identify contextually deceptive ads on a web page. Specifically, we built a detection model that leverages a multi-modal combination of handcrafted and automatically extracted features to determine if a particular ad is contextually deceptive. Evaluations of the model on a representative test dataset and ‘in-the-wild’ random websites yielded F1 scores of 0.86 and 0.88, respectively.

## 1. Introduction

Visual impairment is a relatively common condition that is affecting millions of people worldwide [1]. According to the World Health Organization, at least 2.2 billion people worldwide have near or distant vision impairment and 36 million people are blind [2]. This statistic indicates that 1 out of every 5 individuals in the world has a visual impairment, and 1 out of every 200 people is blind. Despite the significant number of people with blindness, very few assistive technologies are commercially available for these people to conveniently interact with digital web content. One of the most predominant assistive technologies for people with severe vision impairments, including blindness, is a screen reader, such as NVDA (https://www.nvaccess.org/ (accessed on 1 October 2023)), JAWS (https://www.freedomscientific.com/products/software/jaws/ (accessed on 1 October 2023)), or VoiceOver (https://www.apple.com/accessibility/mac/vision/ (accessed on 1 October 2023)).

A screen reader, as the name suggests, narrates the content on the screen and allows blind users to navigate the content using special keyboard shortcuts (e.g., ‘H’ for the next heading). This one-dimensional mode of interaction has been shown to create a plethora of accessibility and usability issues for blind users while interacting with computer applications. These challenges encompass the absence of well-defined structural elements, labels, and descriptions within web elements, hindering the screen reader’s ability to identify and interpret content [3,4,5]. Moreover, the inclusion of dynamic and interactive features, like animations, pop-ups, and videos, may not be screen reader-compatible, resulting in potential interference and incomplete experiences [6]. Additionally, the inconsistency and complexity of web design and layouts can be disorienting and overwhelming for blind users [7]. Furthermore, the lack of feedback and guidance within web applications or websites leaves blind users uncertain and lost, impeding their ability to make informed decisions and navigate effectively [8,9].

Existing works to improve accessibility and usability predominantly focus on general web navigation and visual content, such as images [10,11], and videos [12]; extraneous content, such as advertisements and promotions, however, is still an uncharted research territory. Advertisements and promotions are widely present on most modern websites, serving as a crucial means of generating revenue and frequently offering utility to users. Research indicates that people engage with advertisements by clicking on them and, in general, consider ads as beneficial [13]. However, such extraneous content is, by design, visually rich, and as such, primarily intended for sighted consumption; therefore, it is unclear how these extraneous elements impact the browsing experience and engagement of blind users who can only listen to content using their screen readers. To fill this knowledge gap, we conducted an interview study with 18 blind participants familiar with web browsing.

**Figure 1:**
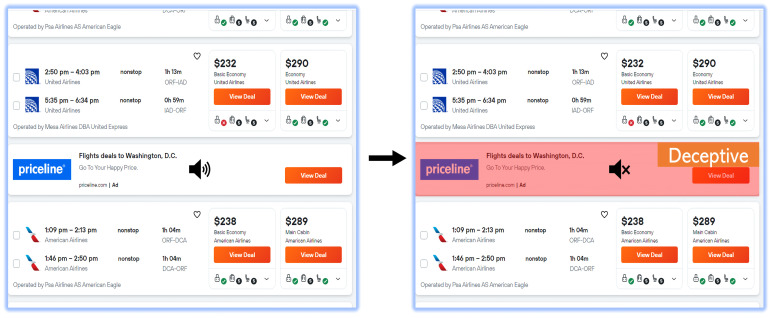
An example of a deceptive ad on a popular *Kayak* travel website. One of the flight results on the list is actually an ad promoting another travel website, namely *Priceline*. The ad location, coupled with the content similarity between the ad and other flights, can potentially deceive blind users due to the limited information provided by their screen readers, e.g., the visual “Priceline” text is read out as just an “image” by a screen reader.

The study uncovered many insights, with the most notable one being that blind users are often ‘deceived’ by ads, specifically the ones that are contextually integrated into the web page content, e.g., native promotion ads that are similar to surrounding web page content, as shown in Figure 1 (https://www.kayak.com/flights/ORF-WAS/2023-12-01/2024-01-11?sort=bestflight_a&attempt=3&lastms=1697423402586&force=true (accessed on 1 October 2023)). The participants further stated that such deceptions often resulted in interacting with content that did not align with their intended objectives or interests, and sometimes the consequences were serious, such as unintentionally installing viruses, revealing personal information, and buying unintended items on shopping websites. Another informative observation from the study was that only a small fraction of the participants used ad blockers; many participants stated that ad blockers were hard to install and configure with screen readers. Moreover, a few participants also expressed that they were unable to access some of their favorite websites with an active ad blocker, as these websites prohibited access to their content on detecting an ad blocker.

To address the problem of contextually deceptive ads, we need an algorithm that can accurately identify such ads on web pages. With such an algorithm, countermeasures (e.g., feeding a screen reader with additional contextual information) can be developed. To devise such an algorithm, we first built an *ad* dataset comprising both deceptive and non-deceptive ads sampled from a diverse set of web pages across multiple domains, including travel, e-commerce, retail, news, hotel booking, and tourism. Leveraging this dataset, we trained a custom multi-modal classification model that uses a combination of both handcrafted and automatically retrieved features as input. Evaluation of the model on a representative test dataset yielded an F1 score of 0.86, thereby demonstrating its effectiveness in distinguishing deceptive ads from non-deceptive ads. We then used this trained classification model in our algorithm to identify all deceptive ads (if any) on any given web page. An ‘in-the-wild’ evaluation of the algorithm on 20 randomly selected websites yielded an F1 score of 0.88. To summarize, our contributions are:

- **In-depth insights into the impact of ads on blind screen-reader users:** Results derived from interviews consisting of a diverse group of 18 blind users provide a deeper understanding of how extraneous content, including ads and promotions, significantly affect the user experience and browsing behavior of blind screen-reader users. This work sheds light on an unexplored aspect of web accessibility and digital inclusion, paving the way for more inclusive ad design on the web.
- **Novel deceptive and non-deceptive ad dataset:** We introduce a dataset comprising both deceptive and non-deceptive ads, meticulously collected from various websites spanning different domains, and manually verified by human experts. This unique dataset serves as a critical resource for researchers and practitioners seeking to explore and address deceptive advertising practices on the web.
- **A novel algorithm for detecting contextually deceptive ads:** We present a novel algorithm with a multi-modal classification model leveraging a combination of handcrafted and auto-extracted features to automatically identify contextually deceptive ads on web pages, and then communicate this information to the users, thereby elevating the quality of blind users’ experiences while fostering a more trustworthy online browsing environment.

## 2. Related Work

Our contributions in this paper build upon prior research in the following areas: (a) non-visual web interaction using screen readers; (b) dark patterns and deceptive web content; and (c) web ad filtering and blocking. We discuss each of these topics next.

### 2.1. Non-Visual Web Interaction Using Screen Readers

A screen reader (e.g., JAWS, NVDA, or VoiceOver) is a special-purpose software that enables visually impaired users to interact with computer applications by listening to their speech output. The prevalence of screen readers among blind users is due to the fact that they are cheaper and simpler to use than hardware devices, like braille displays [14,15,16]. Screen readers provide many shortcuts that facilitate efficient website navigation and content accessibility (https://www.freedomscientific.com/training/jaws/hotkeys/ (accessed on 1 October 2023)) (https://www.nvaccess.org/files/nvdaTracAttachments/455/keycommands%20with%20laptop%20keyboard%20layout.html (accessed on 1 October 2023)). For instance, in NVDA, pressing the ‘D’ key allows users to cycle through and focus on different landmarks, such as headings, navigation menus, or main content areas, providing a quick overview of the page’s organization. Similarly, in JAWS, the ‘D’ key, used in conjunction with a JAWS-specific modifier key, like insert or caps lock, serves the same purpose, aiding users in navigating web pages with ease. Nevertheless, the abundance of shortcuts presents a significant challenge to visually impaired users since it may be arduous to remember and effectively employ each shortcut. For example, NVDA and JAWS offer various complex multi-key combinations for advanced tasks. To trigger these shortcuts, users often need to press multiple keys simultaneously, which can be challenging for individuals with dexterity or motor control issues. An example is the “Insert + 3” key combination in JAWS, which activates the JAWS cursor for advanced navigation and interaction. Remembering and executing such combinations can be daunting, particularly for users with certain physical disabilities or limited dexterity. In general, individuals tend to depend on a limited number of fundamental shortcuts in order to navigate websites [17]. Due to limited shortcut vocabulary, blind users often encounter difficulties navigating modern web pages due to intricate and extensive HTML document object models (DOMs) underlying web pages [18]. In order to tackle these concerns, continuous investigations have been undertaken in the realm of web usability, and the pain points of blind screen-reader users on the web have been identified and addressed [19,20,21,22,23,24,25,26,27,28].

Apart from navigation-related issues, web content itself contains a plethora of visually rich elements, such as videos, images, and memes. Blind users face problems interacting with these elements as they are primarily designed for visual consumption, and often there are no proper textual alternatives for these elements (e.g., alt-text), despite the availability of standard web content accessibility guidelines (WCAG) [29,30]. For example, in the case of images, it has been found that although alt-texts are present, they do not convey the full information equivalent to what a sighted person perceives by looking at these images [31]. To address this issue, AI-based solutions have been recently proposed to automatically generate informative descriptions or captions for visual elements [32]. For instance, Singh et al. [33] built Accessify using machine learning as a means to offer alternative text for every image included on a website, operating inside a non-intrusive framework. Accessify does not necessitate any initial configuration and is compatible with both static and dynamic websites. Apart from images, video accessibility has also gained attention in recent years; e.g., Siu et al. [34] developed a system to automatically generate descriptions for videos and answer blind and low-vision users’ queries about the videos.

In sum, while there is considerable research in the area of web accessibility and usability, to our knowledge, no specific studies have delved into the challenges blind users face when interacting with extraneous content, such as adverts and promotions. This paper aims to fill this research gap.

### 2.2. Dark Patterns and Deceptive Web Content

Brignull first introduced the term “dark patterns ” (https://www.deceptive.design/ (accessed on 1 October 2023)), where he described certain user interface designs as “tricks used in websites and apps that trick you into doing things you didn’t intend to, such as purchasing or signing up for something”. Brignull’s initial work sparked a flurry of academic research that attempted to define and describe dark patterns. Dark patterns—insidious design choices and techniques that manipulate user behavior or deceive users—have become increasingly widespread across various websites [35,36,37,38,39]. In recent years, the prevalence of deceptive practices and dark patterns on numerous digital platforms, including social media, travel websites, e-commerce, apps, and mobile activities, has led to concerns about user trust and autonomy [40,41,42,43,44]. These patterns leverage cognitive biases, create a sense of urgency, or hide essential information, leading users to unintended actions or decisions [35].

While many extant works have focused on different types of dark patterns, there have been relatively fewer specific efforts in the literature regarding deceptive online ads  [45,46]. For instance, Toros et al. [47] focused on deceptive online advertising tactics within e-commerce platforms to shed light on how companies and marketers employ misleading strategies to persuade consumers. They found that companies used tricks to affect the purchasing behaviors of users by incorrectly representing the core products by mimicking, inventing, and relabeling them. Nonetheless, all existing works on dark patterns and deceptive ads [37,39,48,49] have been conducted under the premise of sighted interaction; thus, they do not account for the unique aspects associated with the audio-based interactions of blind users. As ads often contain many visual elements, there is potential for even legitimate ads to be contextually deceptive to blind users in case the screen reader cannot properly communicate these visual elements to the user. For instance, if an ad lacks proper alternative text (alt-text) or fails to communicate the presence of a *Google* ad symbol, visually impaired users may not receive the necessary auditory feedback from screen readers to distinguish between regular content and ads. This lack of visual context can lead to a situation where users may inadvertently interact with or be misled by advertisements that they did not intend to engage with, highlighting the critical importance of ensuring web content is properly labeled and described for accessibility. Therefore, the range of potentially deceptive ads on web pages is wider for blind screen-reader users than for sighted users viewing the same pages, thereby warranting a separate focused analysis to understand the challenges blind users face with ads. We specifically address this need in our work.

### 2.3. Web Ad Filtering and Blocking

Numerous ad detection browser add-ons (e.g., AdGuard (https://adguard.com/en/welcome.html (accessed on 1 October 2023)), AdBlock (https://getadblock.com/en/ (accessed on 1 October 2023)), and AdBlock Plus (https://adblockplus.org/ (accessed on 1 October 2023))) exist to help users avoid ads on web pages. They can be downloaded and installed as browser extensions. They work by blocking communications to ad servers and hiding them from the HTML DOM [50]. They perform this blanket filtering operation by referring to a filter list containing the addresses of all known ad servers along with their pattern-matching rules. However, they do not eliminate any internal promotions or ads [51], most of which are usually deceptive [36]. Internal promotions or ads contribute significantly to the income of many websites and form an integral part of their content. Blocking them could potentially jeopardize the sustainability of these websites and disrupt their layouts.

In addition to commercially available ad blockers, some academic works have proposed ad detection algorithms [52,53]. For instance, Lashkari et al. [54] developed CIC-AB, which is an algorithm that employs machine learning methodologies to identify advertisements and classify them as non-ads, normal ads, and malicious ads, thereby eliminating the need to regularly maintain a filter list (as with earlier rule-based approaches) [51]. CIC-AB was developed as an extension for common browsers (e.g., Firefox and Chrome). Similarly, Bhagavatula et al. [55] developed an algorithm using machine learning for ad blocking with less human intervention, maintaining an accuracy similar to hand-crafted filters (e.g., [51]), while also blocking new ads that would otherwise necessitate further human intervention in the form of additional handmade filter rules. Nonetheless, increasing numbers of websites are now discouraging ad blocking due to the loss of associated ad revenue. Numerous websites have incorporated techniques to identify the existence of ad blockers, potentially leading to the denial of access to content or services upon detection [56].

Complementary to ad blocking, efforts are underway to raise awareness and advocate for the inclusion of accessibility guidelines in the design and implementation of online advertising practices so as to make ads more palatable for all users [57,58]. However, these efforts are still at a nascent stage and may probably require a long time to yield positive outcomes, as in the case of any other accessibility efforts in general, e.g., WCAG guidelines. Moreover, the creators of deceptive ads may not follow ad-related accessibility guidelines for obvious reasons, so there is a need for approaches that detect deceptive ads and communicate their presence and/or details to screen-reader users.

## 3. Understanding Screen-Reader User Behavior with Adverts

To better understand the impact of extraneous ads on interactive behaviors and user experiences, we conducted an IRB-approved interview study consisting of blind people who frequently browse the web.

### 3.1. Participants

We recruited 18 blind participants (11 female, 7 male) for the study through word-of-mouth and local mailing lists. The average age of the participants was 41.7 (Min: 22, Max: 66, Median: 42). The inclusion criteria required the participants to be familiar with screen readers and web browsing. Participants were also required to communicate in English. To eliminate confounding variables, the study excluded people with mild visual impairments, e.g., low-vision users who might use their residual vision to view web content via a screen magnifier and, therefore, do not necessarily require a screen reader. The study also excluded children, i.e., people under the age of 18. All participants indicated that they browsed the web daily for at least one hour. No participant had any physical or aural impairment that restricted their ability to browse the web using a screen reader. Table 1 presents the demographic details of the participants.

### 3.2. Interview Format

The interviews were conducted remotely via Zoom conferencing software. We adopted a semi-structured interview setup where we asked questions about the following topics, specific to interactions with extraneous content.

- *Impact of adverts and promotions on the browsing experience.* Do you use an ad blocker? To what extent do the ads affect your web browsing activity, such as online shopping? What type of ads do you typically come across during browsing? Does the location of an ad on a web page matter? Does your screen reader convey the presence of an ad accurately and provide sufficient details?
- *Browsing strategies and interaction behavior regarding adverts and promotions.* What is your initial reaction or behavior when you encounter an ad while doing a web task? Are there any specific cues, patterns, or elements you specifically consider to determine whether it is safe to select an ad? What strategies do you rely on to recover if you accidentally select an ad?

The participants were additionally encouraged to illustrate their responses live by sharing their screens and demonstrating interaction issues/browsing behavior on their web browsers. The sessions were all recorded, including the live, screen-captured illustrations, with the participants’ formal consent. The personal information collected from the participants is shown in Table 1; no identifiable information was retained after the interview. Each interview lasted about 45 min, and all conversations were in English. To analyze the collected interview data, we used an open coding technique followed by axial coding [59], where we iteratively went over the responses to discover the recurring insights and themes in the data [60] (This method of analysis is common for interview data collected from blind screen reader users.

### 3.3. Results

The qualitative analysis of the participants’ interview responses revealed insights into blind users’ experience with ads on the web. Some of the notable themes that emerged from the interview data will be presented next.

***Ad blockers do not work well with screen readers.*** Most (15) participants indicated that they did not use ad blockers mainly due to one of the following two reasons: (i) They did not know how to install ad blockers to their web browsers using their screen readers; and (ii) they found it difficult to configure ad blockers using screen readers, especially in managing exceptions, for websites that do not permit ad blockers. For the latter case, five participants further stated that they often used to ‘get stuck’ accessing websites since the notification pop-ups for turning off ad blockers (e.g., “ad blocker detected! Please turn off ad blocker and refresh the page”) were inaccessible with screen readers. To avoid this issue, these participants mentioned that they stopped using ad blockers. Only three participants (All ‘Expert’ users) indicated that they frequently used ad blockers and that they knew how to work around website pop-up issues, e.g., B11 said that she opens such websites using a browser’s ‘incognito’ mode, where ad blockers are inactive and, thus, she does not encounter any issues accessing websites.

***Ads often make web activities with screen readers tedious.*** Almost all (16) participants stated that ads increased the amount of time required to finish typical web activities, such as reading news, online shopping, and searching for information. The participants attributed this to the one-dimensional nature of screen reader access, which only allows keyboard-based linear navigation of web content (e.g., ‘H’ key for next heading, ‘TAB’ for next link). Therefore, unlike sighted users who can visually skim through the content and easily avoid ads, blind users have to spend time listening to the content before determining whether it is part of an ad, and then skip it using a series of keyboard shortcuts.

***Screen-reader users frequently encounter three types of ads.*** All participants mentioned that they frequently came across one or more of the following three types of ads: e-commerce deals, member sign-ups, and native promotions ads. However, they also mentioned that the impacts of different types of ads on their browsing experiences were different. All participants mentioned that most e-commerce ads were generally ‘harmless’ if these ads were easily identifiable on web pages, but 10 participants further stated that these ads increased the screen reading time and effort while searching for information online. Regarding membership ads, 8 participants mentioned that they were always wary about providing their personal information online without a sighted friend/family member next to them and, therefore, they avoided selecting these ads. Participant opinions about native-promotion ads were, however, mixed; 4 participants mentioned that such ads were not a concern due to their similarity with e-commerce ads, whereas 7 participants stated that these ads were distracting and sometimes even deceptive given their similarity with the surrounding non-ad web page content.

***Ad location is extremely important for website usability.*** Almost all (16) participants specified that their browsing experiences were affected, to a considerable extent, by the location of ads. Eleven participants specifically mentioned that they were often ‘deceived’ by carefully placed ads that ‘blend in’ with the surrounding content and, therefore, ended up unintentionally selecting them. For example, participant B15 mentioned that there were several ads on travel websites that shared structural similarities with legitimate search result items (see Figure 1); therefore, it was hard to distinguish them from the rest of the surrounding content. B15 further stated that selecting such ads would lead to a significant waste of screen-reading efforts as it always took some time for her to realize that she had unintentionally selected the wrong link.

***Screen readers presently lack the capability to creatively narrate ads to blind users.*** All participants agreed that screen readers presently do not provide enough information about advertisements. During the interview, many participants demonstrated this issue on popular e-commerce websites by sharing their screens. From these demonstrations, the experimenter noted that the screen reader narration of the ads did not match/cover the visual information and cues in the ads, thereby indicating that *what sighted people see in the ads is not the same as what blind people hear about in the same ads from their screen readers*.

***Most screen-reader users’ initial reaction upon encountering an ad is to be careful to avoid accidentally selecting the ad.*** All participants mentioned that they instinctively become ‘extra cautious’ when they encounter an ad, due to their past negative experiences with ads, especially with ones that are contextually deceptive due to their location and content similarity. Twelve participants noted the difficulty in determining the boundaries of an ad while using a screen reader, so they had to carefully listen to content while pressing the navigation shortcuts to determine if they had completely skipped the ad. This sentiment was best expressed by the expert user B5-“*Many advertisements are not just a single image or link that can be easily skipped with a simple shortcut; instead, they are a collection of links, images, and buttons, and accidentally pressing any one of these will result in unintentionally selecting the ad and going to a different website*”.

***To ensure whether an ad is safe to select, screen-reader users mostly rely on sighted friends.*** Two-thirds (6) of the participants explained that they do not select or explore online ads even if they want to, unless they are in the company of their family members or trusted friends. The main reasons provided by the participants for this behavior were safety, privacy, and security concerns. The participants mentioned that they had previously encountered many problems due to deceptive ads, including *unintentionally installing viruses*, *providing personal information*, *buying the wrong shopping products*, and *booking the wrong dates for hotels*.

***Screen-reader users mostly close the browser after realizing they accidentally selected an ad.*** All participants mentioned that they close the browser once they realize that they are exploring irrelevant content related to an advertisement. Four participants further stated that they immediately check if any files are downloaded to their system, and if so, they delete these files.

***Summary.*** The interview study illuminated the blind screen-reader user experiences and behaviors regarding advertisements on websites. One of the main observations was that—due to the limited capability of screen-reader assistive technology—blind users are often deceived by some ads that contextually blend in with the surrounding non-ad content. Blind participants expressed concerns that such deceptions usually lead to unwanted outcomes, such as viruses, privacy breaches, personal information leaks, and unintentional transactions. To address the issue of deceptive ads, we devised a multi-modal deceptive ad detection algorithm, as explained next.

## 4. Deceptive Ad Detection

In this section, we describe our deceptive ad detection method in detail. We first present the overview of our algorithm, including the pseudo-code. Next, we describe the architecture of our multi-modal classification model that leverages both hand-crafted and automatic features extracted from the input ad to determine if the input ad is contextually deceptive or not. Lastly, we provide details on how we trained the model, including the data collection and annotation process.

### 4.1. Algorithm Overview

**Figure 2:**
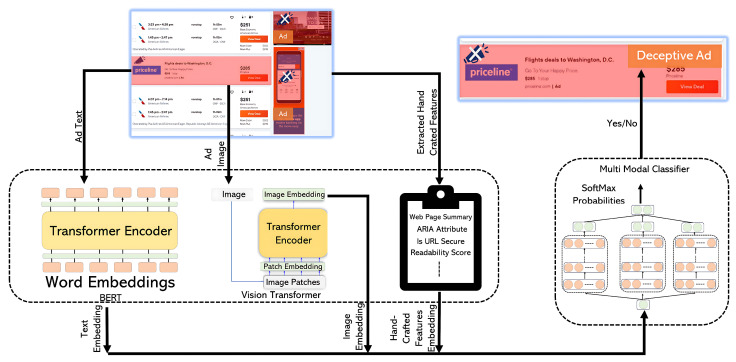
An architectural schematic of the deceptive ad classifier.

Many ads on web pages are not atomic or homogeneous; they are often collections of different HTML elements, including text, images, URLs, buttons, etc. We manually analyzed 50 representative sample web pages from different domains and found that deceptive ads are mostly housed under certain types of HTML DOM nodes (<div>, <iframe>, <script>, <a>, <img>, <span>, and <ins>). The initial stage of the algorithm involves traversing (using depth-first search) the DOM tree and extracting these types of HTML nodes that are potential candidates for deceptive ads. Once we identify potential candidates, we then extract the information from the candidate tags and derive the features. The handcrafted features capture contextual information, such as web page summaries, similarity scores between the web summaries and candidate texts, and candidate URL features. The automatic features capture information from the images and text present within the candidate DOM sub-tree. Most handcrafted features in the dataset are represented as binary values, either a 0 or 1. The automatic features, on the other hand, are represented as numerical values, namely floating point numbers. The second step is to classify the candidates as either deceptive or non-deceptive. Toward this, we trained a custom multi-modal classifier (Figure 2) that leverages a combination of handcrafted and automatically extracted features. All features are then concatenated and fed into the multi-modal classifier (a transformer encoder with an appended LSTM layer). The pseudo-code for the algorithm is detailed in Algorithm 1.
**Algorithm 1:** Detecting dark pattern deceptive ads on a web page
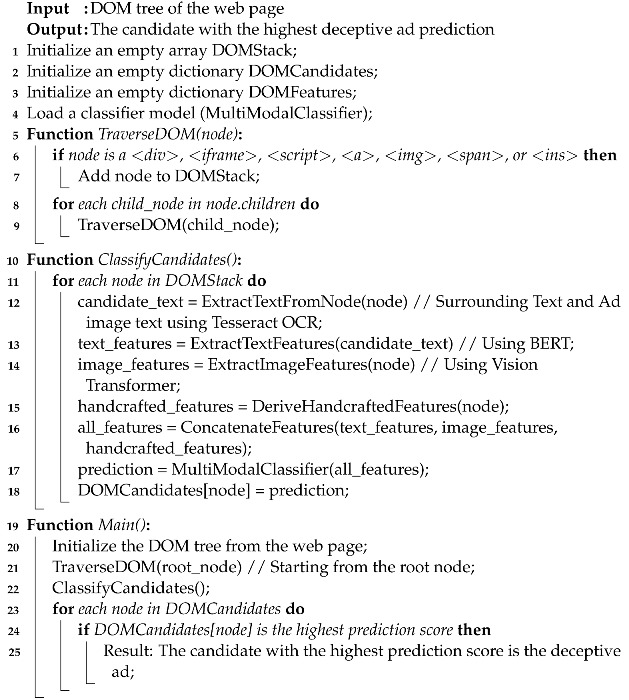


### 4.2. Classification Model

Figure 2 depicts an architectural schematic representing the classification model to identify deceptive ads. The first set of automatic features is derived from the candidate’s ad text, including the text extracted from images in the ad using Tesseract OCR [61]. Specifically, all the text is fed to the pre-trained BERT (BERT-base-uncased) [62] model to extract the features. The second set of automatic features is derived from all the image elements in the ad, using a pre-trained Vision Transformers (google/vit-base-patch16-224) model.

The third set of features is handcrafted after manually analyzing 50 representative sample web pages from different domains, where we observed a few patterns in the deceptive ads. For instance, most deceptive ads did not include ARIA attributes; therefore, screen readers could not provide proper auditory feedback to users. The handcrafted features also includes those that capture the contextual information of the web page, e.g., web page summary, and the similarity between the web page summary and ad text content. The full list of handcrafted features and descriptions are presented in Table 2.

Note that we pre-processed the raw input data before extracting features, as follows. All textual data were cleaned (removing non-printable characters, punctuation, extra spaces, etc.), the image elements were resized to 224 × 224 pixels, and the pixel values were normalized to a common scale ([0, 1]) by dividing all the image arrays by 255. The pre-processed data were then used to extract all the aforementioned automatic features. All extracted features (i.e., image representations, text embeddings, hand-crafted features’ values) were then concatenated and fed to a neural pipeline consisting of 12 transformer encoder layers, followed by an *LSTM* layer and a dense layer. Each encoder layer included a 12 multi-head self-attention mechanism and a feed-forward neural network. The *LSTM* layer included 128 hidden neural units with a *tanh* activation function. In the last dense layer, the *softmax* activation function was used, which took the *logits* as input and converted them into class probabilities. The class with the highest probability (i.e., deceptive or non-deceptive) was then determined to be the output.

### 4.3. Classifier Training

#### 4.3.1. Training Data

***Web page collection***. To train the classification model, we first collected 500 web pages belonging to different domains, including travel sites, news platforms, blogs, e-commerce websites, and more (e.g., *Kayak*, *Priceline*, *Best Buy*, *Tumblr*, and *Reuters*). This diverse selection of domains aimed to capture a comprehensive representation of online content where advertisements are prevalent.

***Web page annotation***. We then manually annotated the deceptive ads on the collected web pages. As mentioned earlier, the deceptive ads are mostly contained in specific DOM nodes (*<div>*, *<iframe>*, *<script>*, *<a>*, *<img>*, *<span>*, and *<ins>*). Therefore, to annotate these ads, we added a custom data attribute *data-deceptive=“true”* to the DOM nodes. For the non-deceptive ads, the value of our custom data attribute was set to false, i.e., *data-deceptive=“false”*.

***Dataset construction***. From the annotated web pages, we first extracted all the deceptive and non-deceptive ads by leveraging the presence of the *data-deceptive* attribute and we then built a supervised dataset (*X*, *y*) by generating all the aforementioned (automatic and hand-crafted) features for representing each ad (i.e., the *X* input variables) and associating each *X* with the target label *y* (1 if deceptive, 0 if non-deceptive). To create a balanced dataset, we sampled 1200 examples or data points from the supervised dataset, containing an equal number (600) of deceptive and non-deceptive ad examples. Our annotated dataset (https://drive.google.com/drive/folders/1UemGmaBLcZ9SWHY0m28Krnc6eyn6OdlV (accessed on 1 October 2023)) and the corresponding code to construct the dataset https://github.com/anonymous66716671/Deceptive-Content/blob/main/Building%20Context%20misleading%20dataset.ipynb (accessed on 1 October 2023), as well as code to extract the features from the dataset (https://github.com/anonymous66716671/Deceptive-Content/blob/main/Deceptive%20URL%20Feature%20Extraction.ipynb (accessed on 1 October 2023)) are all publicly available.

#### 4.3.2. Training Details

For the training process, we utilized an NVIDIA V100 GPU hardware configuration with 128 GB of memory per node. We built the model utilizing the Adam optimizer and a binary cross-entropy loss function, adjusting the learning rate dynamically according to the model’s performance on the validation data. The model was trained across 25 epochs, with a validation split of 0.1. Each epoch consisted of 500 steps. During the model training process, we strategically employed callback functions to optimize the training dynamics. To ensure that we retained the best model configurations, we utilized the *ReduceLROnPlateau* (https://www.tensorflow.org/api_docs/python/tf/keras/callbacks/ReduceLROnPlateau (accessed on 1 October 2023)) callback, which dynamically adjusted the learning rate based on changes in the validation loss. When the validation loss remained stable for two consecutive epochs, the learning rate decreased by a factor of 0.1, facilitating the model’s more efficient convergence toward an optimal solution. The *minimum delta* parameter to the callback was set to 0.0001, ensuring that only substantial improvements were considered and the learning rate was not reduced too frequently. In addition, we assigned a minimum learning rate of 0 through the utilization of the *minimum learning* parameter. This ensured that the learning rate remained above or equal to this given threshold. We integrated the *EarlyStopping* callback, which closely monitored the validation loss and, when necessary, halted training to prevent overfitting. The patience parameter, set to 5, allowed for five consecutive epochs with no validation loss improvement before stopping. The inclusion of carefully designed callback functions played a significant role in the optimization of the model training process, hence improving our ability to identify optimal model parameters.

## 5. Evaluation

In this section, we briefly specify the evaluation process and then present the results for both the offline evaluation of our classifier model on a ground truth testing dataset, and the overall *in-the-wild* evaluation of our algorithm on randomly selected web pages with ads.

### 5.1. Classifier Model Evaluation

As mentioned earlier, we set aside 20% (randomly selected but balanced) from the supervised dataset to test the model’s performance. The test data consisted of examples from different websites from different domains. Standard classification measures (precision, recall, accuracy, and F1-core) were employed to evaluate the performance of the model. We conducted an evaluation of several model configurations on the test dataset (see Table 3): ResNet50 [63] combined with BERT (BERT-base-uncased) [62], both with and without adding features, as well as Vision Transformer (google/vit-base-patch16-224) [64] combined with BERT (BERT-base-uncased) under the same conditions. Our findings reveal that incorporating additional features significantly enhances model performance. The proposed model, equipped with these additional features, outperformed all other configurations, consistently achieving the highest scores, e.g., 0.865 in precision, 0.862 in recall, 0.863 in accuracy, and an F1 score of 0.863. The proposed baseline model also demonstrates strong performance, with scores ranging from 0.794 to 0.800. In contrast, the ResNet50 + BERT and Vision Transformer + BERT combinations exhibit variable performance levels, with marked improvements when features are integrated. These results underscore the importance of feature engineering in optimizing model outcomes. We also conducted an ablation study to examine the contributions of different handcrafted features on the model’s performance; the results are presented in Table 4.

As seen in Table 4, the baseline model includes all the automatic features plus the web page summary and context similarity. We consider this feature set as the baseline as it captures both the ad details and the minimum contextual details that are important to determine if an ad is deceptive, given the web page context. The addition of other contextual features, in general, improves the model’s accuracy from 0.79 (baseline) to 0.86 (with all the features). While we observed this trend of overall improvements in model performance with extra features, there were a few exceptions. For instance, when ζ (no. of URL redirects) was added, there was a tiny drop in performance from 0.865 to 0.863 in the F1 score.

### 5.2. Overall Algorithm Evaluation

We evaluated the overall algorithm’s performance “in-the-wild” by running it on a sample of 20 randomly selected web pages. The web pages were chosen from different domains, including travel, hotel, news, and blogs, in order to provide a representative sample that encompassed different online contexts. The average number of deceptive ads over the 20 web pages was 2. We ensured that the set of 20 selected web pages was disjointed from the dataset we used to train the classifier model so as to evaluate the algorithm’s ability to apply its acquired knowledge to novel and unfamiliar data. The algorithm was evaluated using the standard F1 score metric, which combines precision and recall when assessing performance. The algorithm demonstrated an overall F1 score of 0.88, which is a notable score that highlights the algorithm’s efficacy in identifying deceptive ads across diverse websites.

**Figure 3:**
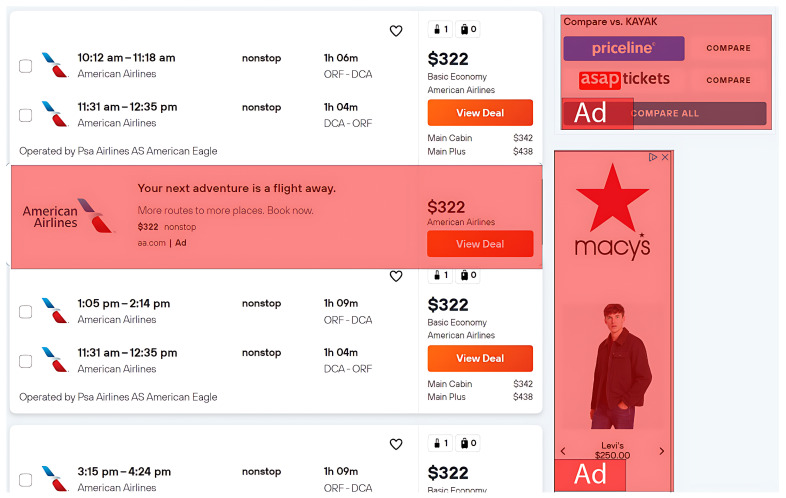
An edge case example of a deceptive ad undetected by the algorithm.

We also analyzed some of the cases where the algorithm failed to detect deceptive ads. For example, in Figure 3, we noticed a deceptive ad placed between the search results with a modified “View Deal” button. The incorrect classification by the algorithm may be attributed to the presence of the ARIA attribute as well as no multiple redirects (observations that are less common in most deceptive ads). For this example, it is likely that these two handcrafted features had more influence on the classification outcome (hence, the error).

## 6. Discussion

While the evaluation demonstrated the algorithm’s overall effectiveness in detecting deceptive ads on web pages, our work had a few limitations. We discuss some of the notable limitations as well as associated future research directions next.

### 6.1. Limitations

Our work was limited to detecting deceptive ads and, therefore, may not generalize to detecting other kinds of dark patterns, such as privacy zuckering, roach motel, confirmshaming, and bait-and-switch [65,66,67]; detecting these dark patterns will likely require novel algorithms. Another limitation was that our overall algorithm was evaluated on a small sample of 20 web pages, constrained by the tedious process of manual annotation. We are in the process of building a larger annotated sample of web pages to facilitate a more detailed and representative evaluation of our algorithm. A third limitation is that our algorithm is just the first step toward addressing the usability issues faced by blind screen-reader users while interacting with web page ads. The next step will be to design front end intelligent interactive systems leveraging our algorithm, which we plan to address in our future research in this area. Lastly, we tested our algorithm on only English-language web pages. Although our algorithm, including the classifier model, has a generic design that easily accommodates other languages, testing on non-English datasets is nonetheless necessary to determine if high performance can be achieved on other language web pages as well.

### 6.2. Bigger Datasets and Alternative Classification Models

In future work, we will aim to expand the size of our datasets to confirm the validity of both our classification model and the overall algorithm. We also plan to expand our algorithm scope by building annotated datasets and training classifiers for other types of dark patterns, such as bait-and-switch, roach motel, etc. For the algorithm models, we will experiment with other deep neural architectures (e.g., PaLI [68], ViLBERT [69]) that have also proven to be effective for multi-modal classification tasks. For automatic image and text feature extraction, we will experiment with different pre-trained models, like BERT-large-uncased [70], and Swin Transformers [71], with methods that enhance the model’s performance [72,73,74].

### 6.3. Downstream Assistive Technology for Non-Visual Ad Interaction

Our algorithm can serve as a foundation for developing novel assistive technologies in browser extensions that can improve blind users’ interactions with online ads. One such technology can replace deceptive ads with screen reader-friendly content by identifying ad elements on a web page, extracting relevant information, and injecting descriptive text alternatives to the detected deceptive ad tag. These text alternatives are then integrated with the screen reader, allowing visually impaired users to understand the content and make informed decisions while browsing. The process enhances accessibility and user safety. Other technology could automatically generate and inject context-relevant textual summaries for ads into the corresponding web page DOM node, so that screen readers can provide more details to the users when they interact with the ad, which can, in turn, help the users make more informed decisions on whether to select or skip the ad. Another idea is to automatically inject informative skip links before deceptive ads in the web page DOM so that a screen-reader user can directly “jump” over the ad content. This strategy will ensure that the user does not accidentally listen to or select the ads under any circumstances without having to filter out the ads. We will explore both of these ideas in future work.

### 6.4. Societal Impact

The significance of web accessibility is paramount to ensuring equal access to digital content for individuals with disabilities, particularly those with substantial visual impairments. Although the primary objective of accessibility is to ensure equal content access for all, it does not inherently ensure optimal interaction usability, i.e., how easily one can interact with digital content. Typically, websites are designed to cater to the needs of sighted users, which might place individuals who rely on screen readers at a disadvantage. In this work, we addressed one such disadvantage involving online ads and promotions. Facilitating more informed interactions with online ads will enable blind screen-reader users to exploit online opportunities and “deals”, avoid performing unintended transactions, prevent accidental downloads, devise efficient web page navigation strategies, and, overall, conduct web activities with fewer security and privacy concerns. This paper took the first step in this regard by devising an algorithm that can automatically identify deceptive ads on web pages. Subsequent downstream efforts can leverage our algorithm to facilitate a more informative interaction with online ads and promotions. The outcomes of these efforts will not only enrich the personal experiences of blind users but will also play a significant role in cultivating a digital environment that is more inclusive and fair, enabling blind individuals to participate more effortlessly in online interactions that were previously arduous and insecure.

## 7. Conclusions

In this paper, we first addressed a knowledge gap regarding how extraneous web content, such as ads and promotions, affects the interaction experience and behavior of blind screen-reader users on the web. Specifically, we conducted an interview study with 18 blind participants, and found that blind screen-reader users are highly susceptible to being misled by contextually deceptive advertisements and promotions. To address this issue, we devised a novel algorithm that is capable of identifying contextually deceptive advertisements on arbitrary web pages. The algorithm was powered by a custom classification model that leveraged a multi-modal set of both hand-crafted and automatically extracted features. When tested on a representative dataset, the model achieved an F1 score of 0.86, whereas an overall “in-the-wild” evaluation of the algorithm on 20 randomly selected web pages yielded an F1 score of 0.88. Given its high performance, we anticipate our algorithm will serve as the foundation for developing future usability-enhancing solutions for informed non-visual ad interactions.

## Figures and Tables

**Table 1:** Demographics of blind participants in the interview study. All information was self-reported.

ID	Age	Sex	Age of Vision Loss	Preferred Screen Reader	Screen Reader Expertise	Web Proficiency	Browsing (Hours/Day)
B1	34	M	Age 10	VoiceOver	Expert	Expert	2
B2	57	M	Do not remember	JAWS	Intermediate	Intermediate	1
B3	45	M	Age 3	JAWS	Intermediate	Intermediate	1
B4	64	F	Do not remember	JAWS	Beginner	Beginner	4
B5	22	F	Age 2	VoiceOver	Expert	Expert	5–6
B6	28	F	Since birth	NVDA	Expert	Intermediate	3–4
B7	45	F	Since birth	VoiceOver	Intermediate	Intermediate	5–6
B8	29	M	Age 5	JAWS	Intermediate	Expert	4
B9	58	F	Do not remember	JAWS	Beginner	Beginner	6
B10	52	F	Do not remember	NVDA	Intermediate	Beginner	2
B11	41	F	Since birth	JAWS	Expert	Expert	1
B12	25	M	Since birth	VoiceOver	Expert	Expert	5
B13	66	F	Do not remember	JAWS	Beginner	Beginner	3–4
B14	32	F	Age 18	NVDA	Expert	Expert	8
B15	48	F	Since birth	NVDA	Beginner	Beginner	2–3
B16	30	F	Since birth	VoiceOver	Intermediate	Intermediate	2
B17	32	M	Age 3	VoiceOver	Expert	Expert	6–8
B18	43	M	Age 8	JAWS	Intermediate	Expert	3

**Table 2:** Handcrafted Classifier features with their descriptions.

Feature	Description
Web page summary	Summary of the web page. All textual information from the web page is fed to the T5 (https://huggingface.co/t5-base (accessed on 1 October 2023)) model to generate the summary.
Context similarity	Cosine similarity score between the ad text and web page summary.
ARIA attribute	Captures the presence/absence of the ARIA attribute in the ad. This feature is set to 1 if present; otherwise, it is set to 0.
Readability score	Flesch readability score (https://simple.wikipedia.org/wiki/Flesch_Reading_Ease (accessed on 1 October 2023)) of ad text ranging from 0 to 100.
Is URL secure	Checks if the ad’s URL is secure, i.e., uses HTTPS protocol. This is set to 1 if the ad’s URL is secure; otherwise, it is set to 0. Our manual analysis shows that deceptive ads rarely use HTTPS in their URLs.
URL hostname	Checks if the ad’s URL has an IPv4 address. This is set to 1 if an ad has a URL with an IPv4 address; otherwise, it is set to 0. Our manual analysis shows that most deceptive ads do not have URLs with an IPv4 address.
URL active/inactive	Checks if the ad’s URL is active. The feature is set to 1 if active, and 0 otherwise. In our manual analysis, we observed that deceptive ad URLs are active only temporarily, i.e., for a short duration.
Number of URL re-directions	Checks if the ad’s URL leads to multiple redirects. This feature is set to 1 if the ad’s URL has multiple re-directions; otherwise, it is set to 0. Typically, a deceptive ad’s URL incurs multiple redirects upon selection.

**Table 3:** Evaluation Metrics.

Model	Precision	Recall	F1 Score	Accuracy
ResNet50 + BERT (without features)	0.66	0.65	0.64	0.64
ResNet50 + BERT (with features)	0.79	0.72	0.71	0.72
Vision Transformer + BERT (without features)	0.79	0.78	0.78	0.77
Vision Transformer + BERT (with features)	0.78	0.79	0.78	0.78
Proposed Model (without features)	0.790	0.800	0.795	0.794
Proposed Model (with features)	0.865	0.862	0.863	0.863

**Table 4:** Model performance, including the ablation study. The baseline model includes all the automatic features plus the web page summary and context similarity. The ablation study focuses on handcrafted features: α = “ARIA Attribute”, β = “Readability Score”, γ = “Is URL Secure”, δ = “URL Host Name”, ϵ = “URL Active/Inactive”, and ζ = “Number of URL Re-Directions”.

Model	Precision	Recall	F1 Score	Accuracy
Baseline model	0.790	0.800	0.795	0.794
Baseline model + α	0.820	0.798	0.809	0.811
Baseline model + α + β	0.818	0.900	0.857	0.850
Baseline model + α + β + γ	0.859	0.870	0.864	0.863
Baseline model + α + β + γ + δ	0.859	0.870	0.864	0.863
Baseline model + α + β + γ + δ + ϵ	0.865	0.865	0.865	0.865
Baseline model + α + β + γ + δ + ϵ + ζ	0.865	0.862	0.863	0.863

## Data Availability

All code and data are available at https://drive.google.com/drive/folders/1cCwBhiFXQrPGcwv3IIlYCh-VbMDcSDGd (accessed on 26 October 2023).

## Acronyms

**Acronyms**	**Expansion**
LSTM	long short-term memory
BERT	bidirectional encoder representations from transformers
ResNet50	residual network 50
HTML	hypertext markup language
DOM	document object model
URL	uniform resource locator
WCAG	web content accessibility guidelines
NVDA	non-visual desktop access
JAWS	job access with speech
